# Supplementary material for: The evolution of same-sex sexual behaviour in mammals
Source: Nat Commun. 2023 Oct 3;14:5719. doi: 10.1038/s41467-023-41290-x (PMC10547684; doi:10.1038/s41467-023-41290-x)
Supplement: Supplementary file 1 — Supplementary Information [file 41467_2023_41290_MOESM1_ESM.pdf]

Supplementary Materials for

The evolution of same-sex sexual behaviour in mammals

This PDF file includes:

Figure S1 to S2
Tables S1 to S9
Supplementary Reference list

Other Supplementary Materials for this manuscript include the following:

Supplementary Data File 1
Supplementary Data File 2

FIGURES

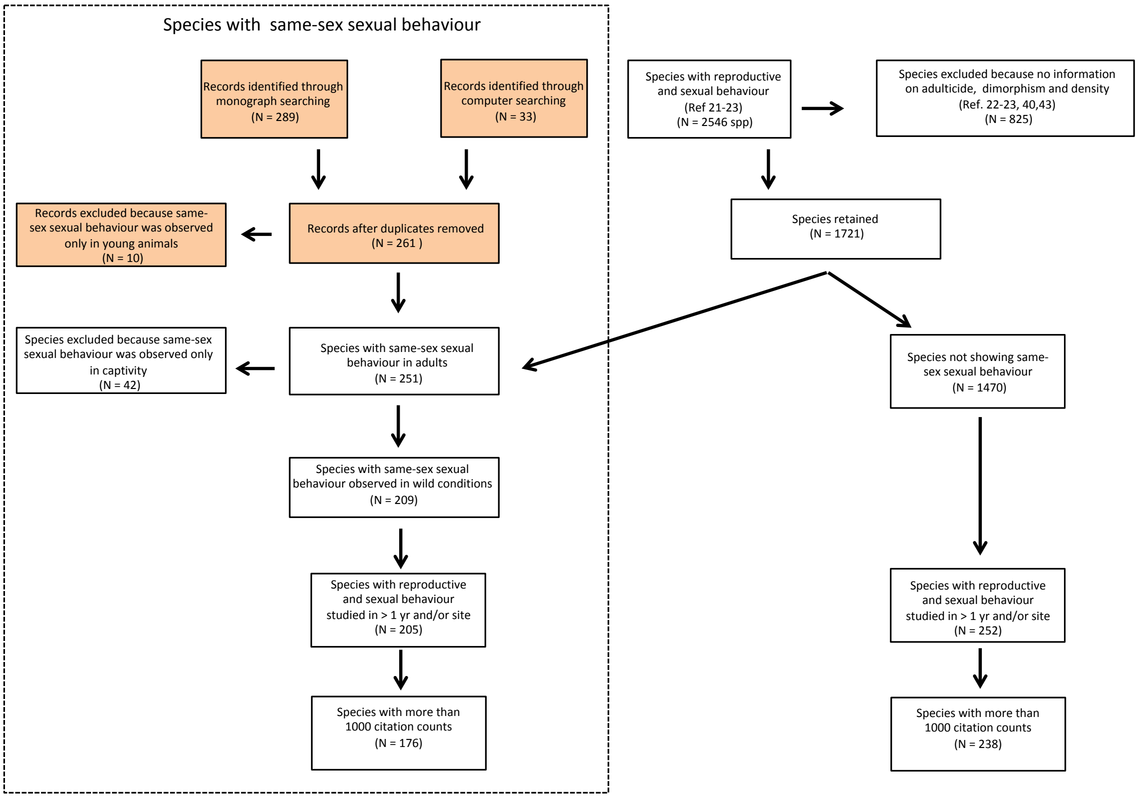

Figure S1. PRISMA flow diagram. The flow diagram depicts the flow of information through the different phases of our review. It maps out the number of records identified (brown squares), included and excluded, and the reasons for exclusions, and the number of species (white squares) included and excluded and the reasons for exclusion.

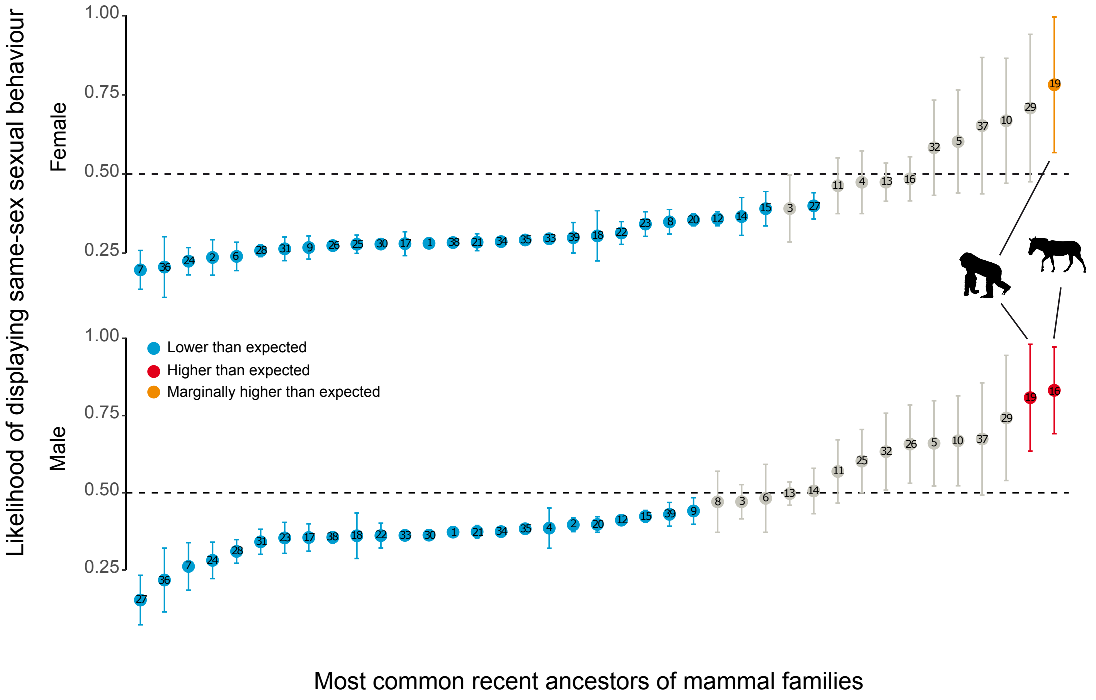

Figure S2. Ancestral reconstruction of same-sex sexual behaviour in mammal families. Likelihood estimation (mean ± 1 standard deviation) across 100 phylogenetic trees of the states of the mrca of main mammal families. Only those families from which same-sex sexual behaviour has been recorded in more than one species and the mrca could be reconstructed are shown. We used for the analysis the subset III. Red dots: significant probability of the ancestor of displaying same-sex sexual behaviour; Orange: marginally significant probability of the ancestor of displaying same-sex sexual behaviour; Blue: significant probability of the ancestor of not displaying same-sex sexual behaviour; Grey: state of the ancestor was equivocal. Numbers indicate the identity of the mammal family as appearing in Table S7. We included the silhouettes of the two families where mrca was significantly higher than expected.

TABLES

Table S1. Number of species included in each of the four subsets used for controlling research intensity. The subsets and methods are described in Methods.

**Subsets**	**With same-sex sexual behaviour**	**Without same-sex sexual behaviour**
**Subset I**: Same-sex sexual behaviour recorded in natural or captivity conditions.	251	1470
**Subset II**: Same-sex sexual behaviour recorded in natural conditions.	209	1470
**Subset III**: Species with reproductive and sexual behaviour studied in > 1 yr and/or site.	205	252
**Subset IV**: Species with more than 1000 citation counts.	176	238

Table S2. Outcome of the analyses testing for phylogenetic correlation between male and female same-sex sexual behaviour for each of 30 phylogenetic randomly-chosen trees tested using each of the four subsets generated to control for research intensity. The table shows the values of AICs of the full models allowing for correlation, the AICs of the models without correlation, the two-sided log-likelihood ratio tests (*LRT*) and their p values.

**Subsets**	AIC full models	AIC no correlation	LRT	P-value
Subset I	13195.5 ± 12.4	13649.1 ± 13.2	461.4 ± 1.4	<0.0001
Subset II	13859.3 ± 19.0	14327.1 ± 20.0	476.9 ± 3.0	<0.0001
Subset III	4260.0 ± 26.6	4424.7 ± 29.0	172.6 ± 5.6	<0.0001
Subset IV	3663.5 ± 16.7	3856.4 ± 19.6	200.9 ± 4.0	<0.0001

Table S3. Average estimation of the phylogenetic signal (D) of both male and female same-sex sexual behaviour (N = 100 randomly-chosen phylogenetic trees) for each subset. Traits evolving under a Brownian model have D = 0, whereas phylogenetic randomness in trait evolution is shown by D = 1. P0 is the significance of the difference between the observed D and D = 0. P1 is the significance of the difference between the observed D and D = 1.

Subset	D	P0	P1
Subset I			
Female	0.440 ± 0.039	0.012 ± 0.002	0.000 ± 0.000
Male	0.635 ± 0.046	0.002 ± 0.000	0.002 ± 0.000
Subset II			
Female	0.595 ± 0.030	0.001 ± 0.000	0.000 ± 0.000
Male	0.629 ± 0.083	0.003 ± 0.000	0.002 ± 0.000
Subset III			
Female	0.588 ± 0.027	0.000 ± 0.000	0.000 ± 0.000
Male	0.639 ± 0.071	0.003 ± 0.000	0.003 ± 0.000
Subset IV			
Female	0.584 ± 0.025	0.001 ± 0.000	0.000 ± 0.000
Male	0.835 ± 0.129	0.002 ± 0.000	0.151 ± 0.264

Table S4. Estimate of the ancestral values of male and female same-sex sexual behaviour for two ancestral nodes of the mammal phylogeny, the ancestor of the mammals and the ancestor of the placentals. Ancestral values were estimated using each subset of species and across 100 randomly chosen phylogenetic trees. Values close to 1 mean that same-sex sexual behaviour was highly likely in that node, values close to 0 mean that same-sex sexual behaviour was probably absent in that node, whereas intermediate values indicate that the presence of same-sex sexual behaviour in those nodes is equivocal.

	Mammals	Placentals
**Female same-sex sexual behaviour**		
Subset I	0.50 ± 0.00	0.08 ± 0.001
Subset II	0.50 ± 0.00	0.32 ± 0.001
Subset III	0.50 ± 0.00	0.32 ± 0.008
Subset IV	0.50 ± 0.00	0.24 ± 0.011
**Male same-sex sexual behaviour**		
Subset I	0.50 ± 0.00	0.10 ± 0.001
Subset II	0.50 ± 0.00	0.40 ± 0.001
Subset III	0.50 ± 0.00	0.40 ± 0.007
Subset IV	0.50 ± 0.00	0.34 ± 0.003

Table S5. Estimate of the gain and losses of male and female same-sex sexual behaviour along the mammal evolutionary history for the four subsets of species. It is shown the median and the 95% confidence intervals within brackets. Note that the number of gains and losses may be larger than the number of species included in the phylogeny because the reconstruction is performed with stochastic mapping, and this method can infer multiple state changes along single branches[1].

	Gains	Losses
**Female same-sex sexual behaviour**		
Subset I	261	296
	[237, 289]	[245, 342]
Subset II	188	234
	[165, 210]	[189, 275]
Subset III	812	813
	[767, 849]	[765, 856]
Subset IV	550	554
	[515, 586]	[511, 592]
**Male same-sex sexual behaviour**		
Subset I	333	373
	[302, 359]	[331, 420]
Subset II	246	291
	[219, 269]	[247, 330]
Subset III	442	449
	[414, 474]	[412, 481]
Subset IV	188	199
	[167, 207]	[173, 226]

Table S6. Estimate of the age in Myr of the clades where same-sex sexual behaviour has evolved (YES) and those where same-sex sexual behaviour has not evolved (NO). Figures are the mean ± sd across 100 phylogenetic trees, and the results of the two-sided t-tests comparing between groups.

	YES	NO	t-test	p-value
**Female same-sex sexual behaviour**				
Subset I	5.9 ± 0.0	7.6 ± 0.1	1.17	0.246
Subset II	5.6 ± 0.3	10.5 ± 0.4	10.45	0.0001
Subset III	5.7 ± 0.3	10.6 ± 0.2	12.39	0.0001
Subset IV	5.7 ± 0.2	13.9 ± 2.5	23.90	0.0001
**Male same-sex sexual behaviour**				
Subset I	5.7 ± 0.1	6.8 ± 0.1	4.49	0.0001
Subset II	6.2 ± 0.2	10.4 ± 0.4	9.73	0.0001
Subset III	6.3 ± 0.2	10.5 ± 0.3	9.84	0.0001
Subset IV	7.6 ± 0.1	14.8 ± 0.2	20.80	0.0001

Table S7. Estimates of the ancestral values of male and female same-sex sexual behaviour for the most common recent ancestors of the mammalian families. Ancestral values were estimated using the subset of species with same-sex sexual behaviour recorded in the wild (subset II). Values close to 1 mean that same-sex sexual behaviour was highly likely in that node, values close to 0 mean that same-sex sexual behaviour was probably absent in that node, whereas intermediate values indicate that the presence of same-sex sexual behaviour in those nodes is equivocal. mean = average ancestral value across 100 phylogenetic trees; sd = standard deviation; z = value of one-sized z-score test testing difference from value = 0.5; p = statistical significance of the z-score test.

		Female				Male			
Code	Family	mean	sd	z	p	mean	sd	z	p1
1	Atelidae	0.281	0.016	13.724	0	0.372	0.017	7.641	0
2	Balaenidae	0.236	0.056	4.702	0	0.396	0.022	4.679	0
3	Balaenopteridae	0.391	0.106	1.033	0.151	0.471	0.099	0.296	0.384
4	Bovidae	0.474	0.099	0.263	0.396	0.385	0.065	1.759	0.039
5	Callitrichidae	0.603	0.163	-0.630	0.264	0.66	0.138	-1.160	0.123
6	Camelidae	0.240	0.045	5.818	0	0.482	0.110	0.164	0.435
7	Canidae	0.197	0.061	4.938	0	0.262	0.077	3.100	0.001
8	Caviidae	0.349	0.039	3.913	0	0.471	0.055	0.523	0.301
9	Cebidae	0.268	0.037	6.337	0	0.441	0.043	1.370	0.085
10	Cercopithecidae	0.668	0.197	-0.852	0.197	0.668	0.146	-1.156	0.124
11	Cervidae	0.463	0.088	0.421	0.337	0.569	0.103	-0.673	0.251
12	Cricetidae	0.359	0.022	6.366	0	0.411	0.013	7.055	0
13	Dasyuridae	0.474	0.060	0.428	0.334	0.497	0.038	0.071	0.472
14	Delphinidae	0.366	0.060	2.259	0.012	0.506	0.073	-0.078	0.469
15	Elephantidae	0.390	0.054	2.017	0.022	0.423	0.019	3.952	0
16	Equidae	0.485	0.070	0.218	0.414	0.832	0.140	-2.365	0.009
17	Felidae	0.28	0.038	5.838	0	0.355	0.044	3.268	0.001
18	Herpestidae	0.305	0.079	2.474	0.007	0.361	0.074	1.894	0.029
19	Hominidae	0.782	0.214	-1.316	0.094	0.808	0.173	-1.779	0.038
20	Hyaenidae	0.355	0.019	7.812	0	0.397	0.025	4.061	0
21	Hylobatidae	0.285	0.027	8.076	0	0.374	0.020	6.199	0
22	Indriidae	0.314	0.036	5.162	0	0.361	0.040	3.446	0
23	Lemuridae	0.342	0.039	4.060	0	0.354	0.050	2.909	0.002
24	Leporidae	0.225	0.043	6.387	0	0.281	0.059	3.717	0

25	Macropodidae	0.278	0.029	7.679	0	0.602	0.102	-0.999	0.159
26	Muridae	0.274	0.011	20.430	0	0.657	0.127	-1.243	0.107
27	Mustelidae	0.399	0.041	2.425	0.008	0.153	0.080	4.338	0
28	Otariidae	0.258	0.019	12.888	0	0.310	0.038	4.962	0
29	Phocidae	0.708	0.233	-0.895	0.185	0.742	0.203	-1.196	0.116
30	Phocoenidae	0.279	0.008	27.839	0	0.363	0.017	8.168	0
31	Procyonidae	0.264	0.037	6.326	0	0.341	0.041	3.908	0
32	Pteropodidae	0.583	0.150	-0.551	0.291	0.633	0.124	-1.069	0.142
33	Rhinolophidae	0.295	0.011	18.302	0	0.362	0.016	8.406	0
34	Sciuridae	0.286	0.008	27.247	0	0.374	0.011	11.16	0
35	Suidae	0.292	0.009	22.527	0	0.383	0.007	15.847	0
36	Tayassuidae	0.206	0.096	3.064	0.001	0.218	0.103	2.737	0.003
37	Trichechidae	0.653	0.215	-0.709	0.239	0.674	0.182	-0.958	0.169
38	Ursidae	0.283	0.009	23.746	0	0.356	0.018	7.864	0
39	Verpertilionidae	0.299	0.048	4.189	0	0.430	0.038	1.818	0.035

Table S8. Estimate of the ancestral values of male and female same-sex sexual behaviour for the main nodes of the clade giving rise to Hominidae. It is shown mean ± 1 sd, and the p-values of the one-sided z-score tests.

Nodes	Male	p-value	Female	p-value
root	0.50±0.00	0.999	0.50±0.00	0.999
Theria	0.38±0.01	0.0001	0.29±0.01	0.0001
Eutheria	0.40±0.01	0.0001	0.32±0.01	0.0001
Primates	0.40±0.01	0.0001	0.31±0.01	0.0001
Haplorrhini	0.40±0.01	0.0001	0.32±0.01	0.0001
Simiiformes	0.45±0.04	0.0001	0.34±0.04	0.007
Catarrhini	0.62±0.11	0.168	0.51±0.14	0.483
Hominidae	0.82±0.12	0.038	0.72±0.10	0.094

Table S9. Outcome of the different analyses testing the effect of sociality and adulticide in presence of male and female same-sex sexual behaviour for the four methods of controlling for sampling effort (see Methods). Significant terms are in bold. Significance is calculated by means of one-sized Z-score, except for the Bayesian analyses (analyses including sampling effort as weighting factor) where significance was calculated by checking whether the confidence intervals include the zero.

	FEMALE				MALE			
	Coeff	Confidence Interval	Z	P	Coeff	Confidence Interval	Z	P
**Sampling effort as covariate**								
**Subset I**								
Phylogenetic signal	0.06	0.04,0.11			0.10	0.05,0.18		
Sociality	**0.65**	**0.27,1.01**	**2.76**	**0.006**	**1.04**	**0.58,1.53**	**3.82**	**0.0001**
Adulticide	-0.37	-1.00,-0.81	0.19	0.313	**0.64**	**0.04,1.22**	**2.18**	**0.029**
S x A	0.24	-0.55,0.84	0.44	0.661	-0.63	-1.32,0.00	1.70	0.089
Sampling effort	**0.87**	**0.81,0.99**	**10.11**	**0.0001**	**0.97**	**0.84,1.81**	**10.59**	**<0.0001**
**Subset II**								
Phylogenetic signal	0.07	0.04,0.17			0.90	0.06,0.16		
Sociality	**1.08**	**0.56,1.54**	**4.01**	**<0.0001**	**1.35**	**0.90,1.94**	**4.66**	**<0.0001**
Adulticide	-0.15	-0.73,0.54	0.36	0.719	**0.72**	**0.21,1.25**	**2.30**	**0.022**
S x A	-0.24	-1.17,0.33	0.39	0.695	-0.76	-1.42,0.09	-1.92	0.054
Sampling effort	**0.95**	**0.81,1.08**	**9.54**	**<0.0001**	**0.92**	**0.82,1.04**	**9.74**	**<0.0001**
**Sampling effort as weighting factor**								
**Subset I**								
Phylogenetic signal	0.96	0.92,0.99			0.96	0.92,0.98		
Sociality	**1.89**	**1.82,2.78**	**NA**	**<0.0001**	**1.70**	**0.78,2.56**	**NA**	**0.0001**
Adulticide	**1.60**	**0.49,2.78**	**NA**	**0.0064**	**2.63**	**1.76,3.52**	**NA**	**0.0001**
S x A	-0.23	-1.88,1.37	NA	0.788	-0.92	-0.98,0.24	NA	0.100
**Subset II**								
Phylogenetic signal	0.97	0.92-0.99			0.96	0.92-0.98		
Sociality	**2.21**	**1.21,3.25**	**NA**	**<0.001**	**1.70**	**0.78,2.69**	**NA**	**<0.0001**
Adulticide	**1.57**	**0.14-2.94**	**NA**	**0.026**	**2.65**	**1.59,3.59**	**NA**	**<0.0001**
S x A	-0.28	-2.28,1.59	NA	0.766	-0.75	-1.93,0.45	NA	0.214
**Sexual behaviour studied profusely**								
**Subset III**								
Phylogenetic signal	0.10	0.06,0.20			0.11	0.07,0,20		
Sociality	**1.16**	**0.65,1.73**	**4.15**	**<0.001**	**1.10**	**0.49,1.64**	**3.58**	**<0.001**
Adulticide	0.69	0.03,1.99	1.80	0.071	**1.20**	**0.54,1.96**	**3.86**	**<0.001**
S x A	-0.63	-1.37,0.78	1.15	0.251	**-0.03**	**-2.18,-0.26**	**2.64**	**0.008**
**Overall behaviour studied profusely**								
**Subset IV**								
Phylogenetic signal	0.05	0.03,0.15			0.08	0.05,0.17		
Sociality	**1.44**	**0.88,2.03**	**4.29**	**<0.001**	**1.28**	**0.64,1.95**	**3.69**	**<0.001**
Adulticide	0.54	-0.18,1.32	1.20	0.229	0.52	-0.16,1.12	1.42	0.157
S x A	-0.64	-1.61,0.37	1.08	0.282	-0.34	-0.08.0.58	0.74	0.457

Supplementary References

1. Huelsenbeck, J. P., Nielsen, R., & Bollback, J. P. Stochastic mapping of morphological characters. *Syst. Biol.* **52**, 131-158 (2003).
