# Supplementary material for: The evolution of same-sex sexual behaviour in mammals
Source: Nat Commun. 2023 Oct 3;14:5719. doi: 10.1038/s41467-023-41290-x (PMC10547684; doi:10.1038/s41467-023-41290-x)
Supplement: Supplementary file 3 — Description of Additional Supplementary Files [file 41467_2023_41290_MOESM3_ESM.pdf]

Description of Additional Supplementary Files

File Name: Supplementary Data 1
Description: List of species with records on same-sex sexual behaviour

File Name: Supplementary Data 2
Description: List of species from which we have information on same-sex sexual behaviour, sociality and adulticide (see Figure S1). Sampling_effort refers to number of citations per species (see Methods)
