# Supplementary material for: The evolution of same-sex sexual behaviour in mammals
Source: Nat Commun. 2023 Oct 3;14:5719. doi: 10.1038/s41467-023-41290-x (PMC10547684; doi:10.1038/s41467-023-41290-x)
Supplement: Supplementary file 6 — Reporting Summary [file 41467_2023_41290_MOESM6_ESM.pdf]

nature portfolio

Corresponding author(s): José M. Gómez

Last updated by author(s): 13/07/2023

Reporting Summary

Nature Portfolio wishes to improve the reproducibility of the work that we publish. This form provides structure for consistency and transparency in reporting. For further information on Nature Portfolio policies, see our Editorial Policies and the Editorial Policy Checklist.

Statistics

For all statistical analyses, confirm that the following items are present in the figure legend, table legend, main text, or Methods section.

n/a	Confirmed	
☐	☒	The exact sample size (*n*) for each experimental group/condition, given as a discrete number and unit of measurement
☒	☐	A statement on whether measurements were taken from distinct samples or whether the same sample was measured repeatedly
☐	☒	The statistical test(s) used AND whether they are one- or two-sided<br>*Only common tests should be described solely by name; describe more complex techniques in the Methods section.*
☐	☒	A description of all covariates tested
☐	☒	A description of any assumptions or corrections, such as tests of normality and adjustment for multiple comparisons
☐	☒	A full description of the statistical parameters including central tendency (e.g. means) or other basic estimates (e.g. regression coefficient) AND variation (e.g. standard deviation) or associated estimates of uncertainty (e.g. confidence intervals)
☐	☒	For null hypothesis testing, the test statistic (e.g. *F*, *t*, *r*) with confidence intervals, effect sizes, degrees of freedom and *P* value noted<br>*Give P values as exact values whenever suitable.*
☒	☐	For Bayesian analysis, information on the choice of priors and Markov chain Monte Carlo settings
☒	☐	For hierarchical and complex designs, identification of the appropriate level for tests and full reporting of outcomes
☒	☐	Estimates of effect sizes (e.g. Cohen's *d*, Pearson's *r*), indicating how they were calculated

Our web collection on statistics for biologists contains articles on many of the points above.

Software and code

Policy information about availability of computer code

Data collection	Data were obtained from the literature and compiled by authors. No software was used for data collection. All data used in this study is provided alongside the manuscript
Data analysis	All analyses were performed with R 4.2.3

For manuscripts utilizing custom algorithms or software that are central to the research but not yet described in published literature, software must be made available to editors and reviewers. We strongly encourage code deposition in a community repository (e.g. GitHub). See the Nature Portfolio guidelines for submitting code & software for further information.

Data

Policy information about availability of data

All manuscripts must include a data availability statement. This statement should provide the following information, where applicable:

- Accession codes, unique identifiers, or web links for publicly available datasets
- A description of any restrictions on data availability
- For clinical datasets or third party data, please ensure that the statement adheres to our policy

The data generated in this study are provided in the Supplementary Information. We downloaded information from:
1) panTheria: https://ecologicaldata.org/wiki/pantheria.
2) Amniote: https://datarepository.wolframcloud.com/resources/Amniote-Life-History-Database

3) EltonTraits 1.0: https://opentraits.org/datasets/elton-traits.html
4) Animal Diversity Web: https://animaldiversity.org/

Human research participants

Policy information about studies involving human research participants and Sex and Gender in Research.

Reporting on sex and gender	NA
Population characteristics	NA
Recruitment	NA
Ethics oversight	NA

Note that full information on the approval of the study protocol must also be provided in the manuscript.

Field-specific reporting

Please select the one below that is the best fit for your research. If you are not sure, read the appropriate sections before making your selection.

☐ Life sciences ☐ Behavioural & social sciences ☒ Ecological, evolutionary & environmental sciences

For a reference copy of the document with all sections, see nature.com/documents/nr-reporting-summary-flat.pdf

Ecological, evolutionary & environmental sciences study design

All studies must disclose on these points even when the disclosure is negative.

Study description	In the manuscript we are currently submitting to Nature Communications we explore the phylogenetic pattern and identify the factors potentially triggering the origin of same-sex sexual behaviour in mammals
Research sample	We included in our study those mammalian species for which same-sex sexual behavior has been recorded and those for which sexual, reproductive and aggresive behaviours have been studied irrespective of the existence of same-sex sexual behaviour.
Sampling strategy	We included the information on same-sex sexual behaviour appearing in several monographs (please, see references indicated in Method section of the manuscript). We complemented this database by conducting computer searches including the terms (alone or in combination, and in British and American spelling) "mammal", "same-sex sexual behaviour", and "homosexual behaviour". In addition, we checked the literature to collect information on the social systems of mammalian species and on their aggresive behaviour.
Data collection	We collected the data when sampling the literature as described above, no information was directly collected from field studies or experiments performed by any of the authors. The three authors sampled the literature and collected the data.
Timing and spatial scale	We started to compile the information on same-sex sexual behaviour in 2019 and finished end of 2021.
Data exclusions	We excluded those mammal species with insufficient information on their sexual, aggresive and social behaviour
Reproducibility	NA
Randomization	NA
Blinding	NA

Did the study involve field work? ☐ Yes ☒ No

Reporting for specific materials, systems and methods

We require information from authors about some types of materials, experimental systems and methods used in many studies. Here, indicate whether each material, system or method listed is relevant to your study. If you are not sure if a list item applies to your research, read the appropriate section before selecting a response.

Materials & experimental systems

n/a	Involved in the study
☒	☐ Antibodies
☒	☐ Eukaryotic cell lines
☒	☐ Palaeontology and archaeology
☒	☐ Animals and other organisms
☒	☐ Clinical data
☒	☐ Dual use research of concern

Methods

n/a	Involved in the study
☒	☐ ChIP-seq
☒	☐ Flow cytometry
☒	☐ MRI-based neuroimaging
